# Ageing and Neurodegenerative Disorders

**DOI:** 10.1155/2015/149532

**Published:** 2015-06-22

**Authors:** Laura Piccardi, Giuseppe Curcio, Liana Palermo, Ming-Chyi Pai

**Affiliations:** ^1^Life, Health and Environmental Science Department, University of L'Aquila, 67100 L'Aquila, Italy; ^2^Neuropsychology Unit, IRCCS Santa Lucia Foundation, 00179 Rome, Italy; ^3^School of Life & Health Sciences, Aston University, Birmingham, West Midlands B15, UK; ^4^Division of Behavioral Neurology, Department of Neurology and Alzheimer's Disease Research Center, National Cheng Kung University Hospital, Institute of Gerontology, College of Medicine, National Cheng Kung University, Tainan 701, Taiwan

The increase in life expectancy and the prevalence of age-related cognitive disorders have led to great interest in studying normal and pathological ageing with the aim of individuating early predictors of degenerative disorders, differential diagnosis, and efficacy of pharmacological and cognitive approaches in the treatment of these disorders. Indeed, considering the great burden of degenerative disorders on national healthcare systems (i.e., in terms of cost and therapy), research aimed at improving the early diagnosis of these pathologies is mandatory. Recent advances in functional imaging methods allow comparative investigation of neurobehavior, neuroanatomy, and neurophysiology pertaining to cognitive and emotional processes in normal and pathological ageing.

A more advanced understanding of underlying mechanisms of ageing and neurodegenerative disorders is desirable for a number of important goals: (1) to enhance the knowledge of their real entity, (2) to evaluate whether the new methods are effective in early and accurate diagnoses, (3) to develop goal-directed training, and (4) to prevent the phenomena through the study of normal ageing mechanisms.

This special issue contains a series of cutting-edge articles that provide innovative information ranging from normal to pathological ageing. Specifically, authors of this issue addressed their discoveries on Parkinson's disease, Alzheimer's disease, and mild cognitive impairment (MCI) also by investigating biological mechanisms that might act as protective factors (i.e., in the work by J. Xu et al.). In line with the importance of early and differential diagnosis as well as that of identifying specific neuropsychological deficits with the aim of improving the current therapies, Y. Ouchi et al. identify the presence of an auditory selective attention deficit in some patients with Alzheimer's disease that helps in predicting their positive response to donepezil therapy. M. S. Maserati et al. discuss their data on Koch's Baum Test in patients with degenerative and vascular dementia and patients with MCI and controls, showing that it is a sensitive tool to early diagnose cognitive impairments. Other studies, such as that by N. Philippi et al., highlight a shift from an initially episodic memory deficit to a semantic memory deficit in the autobiographical memory of Alzheimer's disease which correlates also with the hippocampi volume.

The potential contribution of new techniques and training in improving cognitive symptoms as well as the quality of life in normal and pathological ageing is particularly underlined through this special issue. Specifically, H. L. D. Marra et al. focus on the role of transcranial magnetic stimulation (TMS), a noninvasive brain stimulation technique, in enhancing everyday memory in patients with diagnosis of MCI, suggesting that repeated TMS might be effective as a therapy for MCI which still lacks a specific efficacious therapy. T. Nozawa et al. reported the efficacy of different types of cognitive training on cognitive functions and everyday life activities, such as driving in senior people without cognitive impairments. Finally, concerning the quality of life and psychosocial functioning of dementia caregivers, S. K. Trapp et al. provide a fundamental contribution in demonstrating associations between personal strengths and enhanced psychosocial functioning of dementia caregivers in the specific context of Latin America.

